# Development of a Network of Accurate Ozone Sensing Nodes for Parallel Monitoring in a Site Relocation Study

**DOI:** 10.3390/s20010016

**Published:** 2019-12-18

**Authors:** Brandon Feenstra, Vasileios Papapostolou, Berj Der Boghossian, David Cocker, Andrea Polidori

**Affiliations:** 1 South Coast Air Quality Management District, Air Quality Sensor Performance Evaluation Center (AQ-SPEC), Diamond Bar, CA 91765, USA; vpapapostolou@aqmd.gov (V.P.); bderboghossian@aqmd.gov (B.D.B.); 2 Department of Chemical & Environmental Engineering, University of California-Riverside, Riverside, CA 92521, USA; dcocker@engr.ucr.edu; 3 Bourns College of Engineering, Center for Environmental Research and Technology (CE-CERT), University of California-Riverside, Riverside, CA 92507, USA

**Keywords:** ozone, sensor network, sensor node, mountain community monitoring, parallel monitoring, site relocation study

## Abstract

Recent technological advances in both air sensing technology and Internet of Things (IoT) connectivity have enabled the development and deployment of remote monitoring networks of air quality sensors. The compact size and low power requirements of both sensors and IoT data loggers allow for the development of remote sensing nodes with power and connectivity versatility. With these technological advancements, sensor networks can be developed and deployed for various ambient air monitoring applications. This paper describes the development and deployment of a monitoring network of accurate ozone (O_3_) sensor nodes to provide parallel monitoring in an air monitoring site relocation study. The reference O_3_ analyzer at the station along with a network of three O_3_ sensing nodes was used to evaluate the spatial and temporal variability of O_3_ across four Southern California communities in the San Bernardino Mountains which are currently represented by a single reference station in Crestline, CA. The motivation for developing and deploying the sensor network in the region was that the single reference station potentially needed to be relocated due to uncertainty that the lease agreement would be renewed. With the implication of siting a new reference station that is also a high O_3_ site, the project required the development of an accurate and precise sensing node for establishing a parallel monitoring network at potential relocation sites. The deployment methodology included a pre-deployment co-location calibration to the reference analyzer at the air monitoring station with post-deployment co-location results indicating a mean absolute error (MAE) < 2 ppb for 1-h mean O_3_ concentrations. Ordinary least squares regression statistics between reference and sensor nodes during post-deployment co-location testing indicate that the nodes are accurate and highly correlated to reference instrumentation with R^2^ values > 0.98, slope offsets < 0.02, and intercept offsets < 0.6 for hourly O_3_ concentrations with a mean concentration value of 39.7 ± 16.5 ppb and a maximum 1-h value of 94 ppb. Spatial variability for diurnal O_3_ trends was found between locations within 5 km of each other with spatial variability between sites more pronounced during nighttime hours. The parallel monitoring was successful in providing the data to develop a relocation strategy with only one relocation site providing a 95% confidence that concentrations would be higher there than at the current site.

## 1. Introduction

### 1.1. Ozone Pollution

Ozone (O_3_) is a highly reactive gas that is comprised of three oxygen atoms. In the stratosphere (10–50 km above the earth’s surface), O_3_ is generated naturally and provides a protective layer that shields the earth from harmful ultraviolet (UV) rays emitted by the sun. In the troposphere (0–10 km above earth’s surface), O_3_ is considered an air pollutant and harmful to public health and the environment. The effects of O_3_ on human health include reducing lung function and irritation of the respiratory system. Increases in exposure to O_3_ have been associated with increases in school absenteeism [1,2,3] and increases in the risk of death from respiratory causes [4,5,6,7]. In a long-term study on children, reductions in air pollutants have been associated with statistically significant decreases in bronchitis symptoms like asthma [8,9]. High concentrations of O_3_ have been recognized as a phytotoxic threat to forests, crops, and vegetation [10,11].

Tropospheric O_3_ or ground-level O_3_ is formed by chemical reactions between oxides of nitrogen (NO_x_) and volatile organic compounds (VOC) that occur in the presence of sunlight. This process is known as the photolytic cycle and is shown in Equations (1)–(3) [12]. Prime conditions for generating O_3_ typically occur during the summer months when intense sunlight is coupled with mobile and stationary sources emitting carbon monoxide (CO), VOC, and NO_x_. Without sunlight, photolysis of nitrogen dioxide (NO_2_) in Equation (1) ceases and Equation (3) leads to the removal of O_3_ from the atmosphere when fresh emissions of NO are present. Commuter traffic in the late afternoon and early evening typically provides a source of fresh NO emissions leading to O_3_ titration. In rural communities, fewer sources of NO may cause less titration of O_3_ by NO which may lead to higher nighttime O_3_ concentrations than nearby urban environments [13].

(1) $${\mathrm{NO}}_{2}+\mathrm{hv}\;\to \mathrm{NO}+O\cdot$$

(2) $$O\cdot +O_{2}\to O_{3}$$

(3) $$O_{3}+\mathrm{NO}\to {\mathrm{NO}}_{2}+O_{2}$$

### 1.2. Regulation

In the United States, O_3_ concentration levels are regulated by the United States Environmental Protection Agency (U.S. EPA) under the Clean Air Act (CAA). The U.S. EPA establishes National Ambient Air Quality Standards (NAAQS) for criteria pollutants which include CO, lead (Pb), NO_2_, O_3_, particulate matter (PM), and sulfur dioxide (SO_2_). The State of California further regulates these pollutants with the California Ambient Air Quality Standards (CAAQS) established by the California Air Resources Board (CARB). These standards are designed to protect public health and the environment. The latest federal and state standards for O_3_ are shown in Table 1.

### 1.3. Ozone Levels in the South Coast Air Basin and Monitoring in the San Bernardino Mountains

The South Coast Air Quality Management District (South Coast AQMD) is the air pollution agency for the South Coast Air Basin (SCAB) which is in Southern California and includes all of Orange County and the urban portions of Los Angeles, Riverside, and San Bernardino Counties. In order to determine regional attainment for ambient air quality standards, South Coast AQMD operates a network of air monitoring stations (AMS) equipped with EPA approved instrumentation that measures criteria air pollutants across the basin. South Coast AQMD operates 29 Federal Equivalent Method (FEM) O_3_ instruments. Significant improvement has been achieved in reducing O_3_ while population, vehicle miles traveled, economic activity, and goods movement in the region has been increasing. Large emissions of O_3_ precursors (NO_x_ and VOCs) along with the topography and meteorology of the region lead to some of the worst O_3_ pollution in the nation [14]. The San Bernardino Mountain (SBM) Communities (SBMCs) are especially at risk for severe O_3_ episodes as polluted air travels inland with onshore wind from Los Angeles. Regional-scale temperature inversions that occur below the heights of the mountain crests lead to stagnant air conditions while clear skies and abundant sunlight provide conditions conducive for O_3_ formation [15]. In 2015, one or more of the South Coast AQMD’s O_3_ reference analyzers exceeded the most current federal standard (2015 8-h NAAQS: 70 ppb) on 113 days. Of the top ten monitoring sites in the nation for most frequently exceeding the 8-h standard, seven are located within the SCAB. Monitoring sites within the San Bernardino County exceeded this standard 102 times in 2015 with the Central San Bernardino Mountains air monitoring site (Crestline AMS) exceeding the 8-h O_3_ standard 86 times; more than any other O_3_ monitoring location in the basin. The maximum 8-h average O_3_ concentration recorded in the SCAB in 2015 was measured at the Crestline AMS at 127 ppb (AQMP, 2016).

The main goal of this study is to determine if a relocation site in a nearby community would experience the same or similar O_3_ profile to the current monitoring site by testing the hypothesis that O_3_ concentrations in nearby communities are consistent spatially and temporally. While O_3_ is a secondary pollutant that is formed by reactions between primary pollutants (NO_x_ and VOC) in the presence of sunlight and is often considered a regional pollutant, a recent community level O_3_ monitoring campaign in Riverside, CA found that O_3_ concentrations vary spatially across a community [16]. In early 2017, South Coast AQMD was faced with the potential need to relocate the Crestline AMS due to uncertainty that the lease agreement would be renewed. If the lease was terminated, circumstances may prevent the option to perform parallel monitoring or perform parallel monitoring during the high O_3_ season, typically occurring from July through September. At a minimum, parallel monitoring must be conducted during the season when maximum concentrations are expected [17]. Since this monitoring station experiences some of the highest O_3_ concentrations in the basin, parallel monitoring at the current and potential relocation monitoring sites was determined to be necessary to develop an appropriate relocation strategy. While parallel monitoring is not required via statute or regulation when relocating a monitoring site, not performing parallel monitoring may have regulatory consequences if the relocation site does not meet the same monitoring objectives of the current monitoring location. Parallel monitoring provides a mechanism to determine if the relocation site can meet the current monitoring objectives.

The current monitoring objectives of the Crestline AMS include evaluation of ambient air quality data, protection of public health, development and evaluation of control plans, and air quality research. The evaluation of ambient air quality provides data to determine the attainment of ambient air quality standards (NAAQS and CAAQS), assess progress in achieving standards, and track long term trends. The protection of public health is achieved through communicating the Air Quality Index (AQI) results to the public in a timely manner and documenting population exposure to air pollutants [18]. Data used for research involves long-term trend analysis and tracking impacts on the environment and the public health effects of air pollutants. Parallel monitoring can also provide insights into the continuity of measurements between an old and new monitoring site. Continuity of measurements in one location is ideal for tracking long term trends for assessing progress in achieving and maintaining national and state standards, developing and evaluating State Implementation Plans (SIP) for attaining the standards, and providing long term data repositories for answering questions posed by researchers. The South Coast AQMD has been monitoring O_3_ in Crestline, California since 1973 and maintaining the continuity of measurements is ideal for long-term trend analysis [19].

This study aims to investigate the spatial and temporal variability between Crestline and the three potential relocation sites by parallel monitoring during the high O_3_ season. With the expected results impacting the relocation strategy of a monitoring site with high O_3_ concentrations, the monitoring project required an accurate, precise, and reliable O_3_ sensor that could be deployed in remote mountain locations with power and connectivity versatility.

### 1.4. Evaluation of Ozone Sensing Technology

In 2014, the South Coast AQMD established the Air Quality Sensor Performance Evaluation Center (AQ-SPEC) to evaluate the performance of consumer and research-grade sensors against federally approved instrumentation. AQ-SPEC evaluates gas-phase and particle-phase sensors under both ambient field and controlled laboratory conditions. Results from these performance evaluations are publicly available on the AQ-SPEC website at www.aqmd.gov/aq-spec. The methodology of low-cost sensors that measure O_3_ is typically categorized as either metal-oxide or electrochemical methods. The performance of low-cost gas-phase sensors can be impacted by changing environmental factors (e.g., temperature and humidity), long-term drift, and interfering pollutants [20,21,22,23]. Electrochemical sensors for O_3_ detection often experience inference from other oxidizing gases commonly found in ambient environments [20]. When deployed for ambient air monitoring, the electrochemical O_3_ sensors are often coupled with a NO_2_ sensor in order to subtract out interference from local NO_2_ concentrations. While metal-oxide O_3_ sensors are selective to O_3_, previous deployments of this technology have shown reduced sensitivity to O_3_ concentrations over time in extended field deployment studies [24,25].

AQ-SPEC evaluated the 2B Tech Personal O_3_ Monitor (POM, 2B Technologies, Boulder, CO, USA) with the field and laboratory evaluation results indicating that the POM is capable of accurate and precise O_3_ measurements. The POM is a miniature UV-absorption based monitor that uses a folded optical path (“U” shaped) to achieve a path length similar to that used in a regulatory-grade O_3_ instrument that is designated as U.S. EPA FEM [26]. In August of 2015, the UV absorption methodology used in the POM was designated by the U.S. EPA as FEM for O_3_: EQOA–0815–227. In the two-month AQ-SPEC field evaluation, the coefficient of determination (R^2^) for a triplicate set of POMs was found to be 1.0 with a mean absolute error (MAE) less than 2 ppb [27,28]. In the AQ-SPEC laboratory evaluation, the performance of the POM was found not to be adversely affected by the NO_2_ interferent or extreme environmental conditions (i.e., high/low temperature and relative humidity) [28,29]. In a previous study to monitor O_3_ for the Hong Kong Marathon, the POM was selected due to its ability to measure O_3_ without interferences from common oxidizing pollutants found in ambient air [30].

## 2. Materials and Methods

### 2.1. Node Design and Development

Based on project monitoring requirements, the POM was selected as the O_3_ sensor to be incorporated into the sensing node. The POM weighs 0.3 kg with dimensions of 10 × 7.6 × 3.8 cm and is shown in the Supplementary Materials Figure S1. The POM is powered by 12-volt direct current (DC) and integrates well into battery, solar, or plugin (AC/DC converter) applications. Particular to this study, the POMs were equipped with a particulate filter at the sample inlet to prevent dust and aerosols from reaching the sensor optics. In contrast to many of the commercially available O_3_ sensors that use a fan or passive sampling, the sampling mechanism of the POM is a small pump that controls sample flow through the unit. The pump is one of the factors affecting the commercial price of the device. Since only a small network of three sensors would be deployed, the cost was not a primary concern in sensor selection for this monitoring application. Enough monetary resources or access to loaning such sensors via a sensor library program would be required for other researchers to deploy similar types of sensor networks.

Due to the timeline requirements to build and deploy a network of O_3_ monitors in the region during the high O_3_ season, the decision was made to build a sensor network that would be easily deployed in contrast to deploying additional monitoring stations that would require constructing, building, and siting three additional ambient air monitoring shelters equipped with FEM O_3_ analyzers, zero air generators, and gas calibrators. Constructing, building, and siting additional monitoring stations with required equipment would have been time and cost-prohibitive to meet project timeline requirements to monitor O_3_ in the region during the high O_3_ season.

The POM is not an “Internet of Things” (IoT) connected device. Out of the box, POM data can be stored internally on the POMs internal memory (limited to ~6 days for 1-min averaged data) and/or data can be transmitted over a USB or serial port for logging data externally with a data acquisition solution. The POM was coupled with an IoT communications device for data acquisition, edge data processing, and data telemetry to a cloud-based platform for data storage and visualization. A remote IoT monitoring solution was selected, which included data acquisition hardware (i.e., model Thiamis 1000 (T1K), Netronix Inc., Philadelphia, PA, USA) and a cloud-based environmental monitoring software with web-based application functionality that provides access to real-time and historical monitoring data (i.e., Environet, Netronix Inc., Philadelphia, PA, USA). The T1K is equipped with both cellular and Wi-Fi data communication, a real-time clock, and 8 GB memory (see Supplementary Materials Figure S2). The real-time clock and internal memory allowed the T1K to continue recording data even if the data connection (Wi-Fi or cellular) was intermittent or unavailable for an extended period. The three POMs were configured to output data every 10 s. The 10-s data was transmitted to the T1K through the serial to 3.5 mm cable provided by 2B Technologies. The T1k recorded and performed edge data processing to average the 10-sec O_3_ values to 1-min average O_3_ concentrations and thus reduced the data transmission rate to 1/6 of the original data output from the sensor. These 1-min O_3_ concentrations were then transmitted from the T1K via a cellular or Wi-Fi network to Environet for data storage and visualization. The 1-min data output was selected for this monitoring network to allow the POM data to be time-matched with the output of the regulatory air monitoring station FEM ozone instrumentation for pre-deployment collocation calibration purposes.

**Figure 1:**
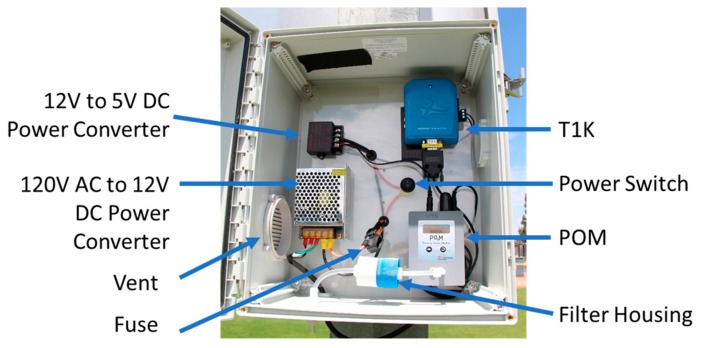
Ozone sensor node with labeled components.

The T1K and the POM were housed in a weatherproof polycarbonate enclosure (Fibox, Glen Burnie, MD, USA) with dimensions of 35.5 × 30.5 × 17.8 cm. The box was fitted with the appropriate backing plate for mounting the components in the enclosure which allowed for easy access to remove hardware from the enclosure for potential repairs or replacement. Two vents were installed in the box for heat dissipation and to ensure that the sampling of the POM was not pumping air into a leak-tight box. The sensor node was powered via a 120/12V AC to DC power converter. The node could be optionally configured for solar power by adding a 12 V battery, 50 W solar panel, and charge controller. The total cost per node is roughly $6500 USD. The bill of materials (BOM) for the sensor node is shown in Supplementary Materials Table S1. Figure 1 shows the sensing node with the major components labeled. The result of this development was an accurate O_3_ sensing node that could be successfully deployed in rural communities with varied access to power and connectivity to transmit real-time data and visualize data remotely.

### 2.2. Nodes’ Deployment

Three O_3_ sensor nodes were constructed and deployed for this parallel monitoring application. Prior to deployment, the POMs were calibrated against a calibration transfer standard (CTS) at South Coast AQMD headquarters in Diamond Bar, CA. The CTS, Thermo Scientific Model 49i O_3_ analyzer, was connected to a manifold along with the three POMs. The manifold was then inundated with varying O_3_ concentrations by an O_3_ generator. The POMs were calibrated with the in-line particulate filter upstream of the sampling inlets to ensure that the calibration configuration matched the deployment configuration. The initial calibration was a 2-point calibration with a zero and span at 250 ppb of O_3_. The calibration parameters, slope (S) and offset (Z), derived from the 2-point calibration were inputted into the POM via the POM’s user interface as outlined in the 2B Technology operational manual [31]. After calibration, the POMs were verified against the CTS with ramping O_3_ in the following sequence: 0, 250, 200, 150, 100, and 50 ppb (see Supplementary Materials Figure S3a–c). The slopes ranged from 0.98 to 1.00 with R^2^ values greater than 0.99. The intercepts ranged from 0.3 to 2.1 ppb. The results of the verification indicate that the POMs accurately and precisely measured O_3_ over a wide range of concentrations that were inclusive of ambient levels not exceeding 200 ppb during the study.

The experimental deployment design incorporated three phases: pre-deployment co-location, deployment, and post-deployment co-location. The three phases of deployment are summarized in Table 2. The pre- and post-deployment co-location took place at the Crestline AMS, which is equipped with a FEM O_3_ reference analyzer (model 49i, Thermo Scientific, Waltham, MA, USA). The pre-deployment co-location at the AMS allowed for the implementation of an in-situ field calibration of the 3 POMs to the station reference analyzer. The post-deployment co-location at the AMS allowed for a verification of POM performance at the conclusion of the study in order to verify the in-situ field calibration and the deployment results.

**Figure 2:**
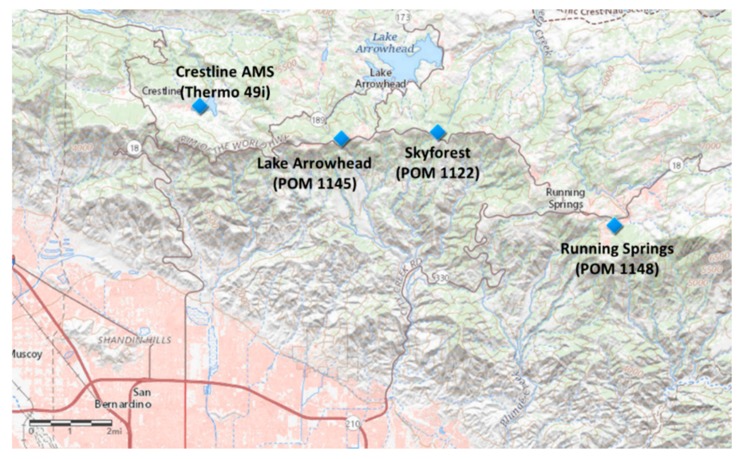
Map of Eastern San Bernardino Mountain Communities and deployment locations.

The three additional deployment locations were selected based on their potential to serve as a possible relocation site for the current Crestline AMS. The deployment locations are shown on a map of the San Bernardino Mountains in Figure 2 (larger extent map in Supplementary Materials Figure S4). The three additional locations are on the south slope of the San Bernardino Mountains and located near the California State Route 18 (SR-18). SR-18 begins in San Bernardino at State Route 210 (SR-210) and travels to Big Bear City and then out to the high desert region near Victorville and Interstate 15 (I-15). SR-330 which also originates in San Bernardino and merges with SR-18 in Running Springs.

### 2.3. Data Processing and Analysis

When examining the POMs during the co-location time periods, ordinary least squares (OLS) regression statistics along with mean bias error (MBE) and MAE were utilized to characterize the POMs performance against the Thermo 49i O_3_ measurements from the Crestline AMS. Information on the measurement error calculations and equations for MBE and MAE can be found in the Supplementary Materials Equations (S1) and (S2).

When examining the POMs and the Crestline Thermo 49i during the deployment periods, OLS regression statistics and bias deviations between Crestline and the three alternative locations were utilized to characterize spatial and temporal differences between sites. Equations for mean bias deviation (MBD) and mean absolute deviation (MAD) are found in Supplementary Materials Equations (S3) and (S4). The MBD between the relocation sites and the Crestline location provides a metric that indicates the tendency of a relocation site to either under- or over-estimate O_3_ concentrations when compared to the Crestline AMS location. The MBD is a relative measure that can be either positive or negative based on whether the relocation site under- or over-estimates O_3_ concentrations when compared to the Crestline location. Care should be taken when examining the MBD since the positive and negative errors will cancel each other out. The MAD provides a better metric for examining the absolute deviations between the Crestline location and potential relocation sites.

The 1-min data collected during the deployment phase was calibrated according to the OLS calibration factors derived from the pre-deployment co-location period. The 1-min data was processed to remove negative and extremely high concentrations (> 250 ppb) from the data set. The 1-min data was then run through a Hampel outlier detection algorithm to remove and replace temporal outliers (see Supplementary Materials Section S7). The rolling Hampel filter compares each data point to a rolling median value of the last 10 consecutive data points in a data series. A threshold of six standard deviations was used to characterize a value as an outlier and replace it with the rolling median value. The cause of the outliers may have been power surges or temporary glitches with the POM or data transmission. Data were then averaged to 1-h mean O_3_ concentrations with a requisite of 42 or more 1-min data points to generate a valid 1-h mean O_3_ concentration. These 1-h averages for the three POMs and Crestline reference monitor were then matched on date and time to enable the parallel monitoring comparisons between the reference site and three relocation sites. Any row with a missing concentration value for either Crestline or the three relocation sites was removed from the analysis so the four locations could be compared across a complete matching data set.

In following the CARB Air Monitoring Technical Advisory Committee (AMTAC) document providing guidelines for site relocation and parallel monitoring, a data set of high values was created by finding the daily maximum 1-h O_3_ concentration for each location and then filtering to keep values that exceed a threshold value. The threshold value was set at 87.4 ppb which represents the top 20% of the prior three years of daily maximum 1-h O_3_ concentrations collected at the Crestline AMS. From this data set, MBD could be calculated to determine if a relocation site would be higher or lower than the current monitoring site with calculating the upper and lower limits of the 95% confidence interval (CI) on the MBD. Calculations for the 95% CI on the MBD have been adapted from the CARB’s Guidelines for Parallel Monitoring [17] (Supplementary Materials Equations (S5)–(S8)).

## 3. Results and Discussion

### 3.1. Pre-Deployment Co-location Period at Crestline

**Figure 3:**
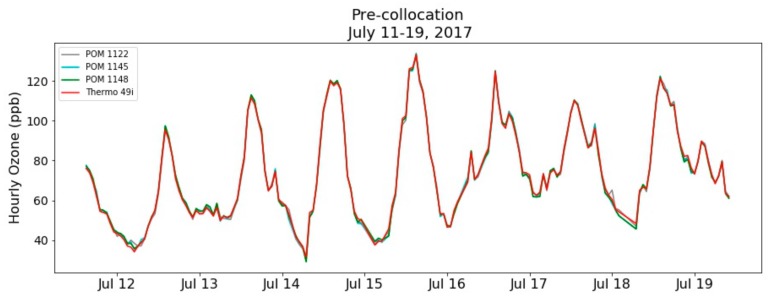
Pre-deployment co-location at Crestline time series for 1-h mean O_3_ concentrations after the in-situ field calibration was performed.

**Figure 4:**
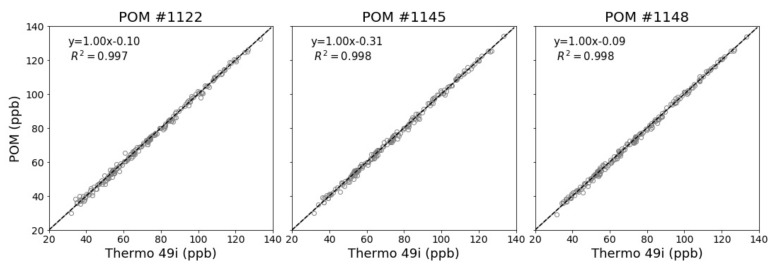
Pre-deployment co-location correlation plots for 1-h O_3_ concentrations after the in-situ field calibration was performed.

Data collection for the pre-deployment co-location at the Crestline AMS took place from 11 July to 19 July 2017, which provided for nearly eight days of co-location data. During the pre-deployment co-location, ambient temperature ranged from 16 to 30 °C with a mean temperature of 23.4 ± 3.3 °C and ambient relative humidity (RH) ranged from 21% to 70% with a mean RH of 46.4% ± 11.2%, as measured by the AMS meteorological equipment (model HC2-S3, Rotronic, Hauppauge, NY, USA). During these eight days, the range of 1-min O_3_ concentration was 110 ppb with a maximum of 141 ppb measured on 15 July, as recorded by the Crestline AMS FEM O_3_ instrument. The 1-min datasets from the POMs and FEM were filtered for values < −5 ppb and > 250 ppb. The 1-min data was then time-matched and OLS regression analysis was performed for the POMs against the Thermo 49i reference analyzer to perform an in-situ field calibration. The co-location OLS calibration offsets for the POMs were small with slope offsets < 0.07 and intercept offsets < 1.6 ppb (see Supplementary Materials Table S2). The in-situ field calibration is effective in correcting for slope and intercept offsets and reducing the MBE between the POMs and the Thermo 49i. The MAE calculated for the three POMs at the 1-min time interval is <4 ppb. Due to the inherent fluctuations of 1-min data points, the MAE was not effectively reduced by the in-situ field calibration. By averaging to 1-h mean O_3_ concentrations, the MAE between the three POMs and the Crestline O_3_ monitor was reduced to less than 1 ppb. Figure 3 shows the time-series for the pre-deployment co-location time period with Figure 4 showing the correlation plots of the POMs against the Thermo 49i after the in-situ field calibration was performed. The low measurement error of the POMs against the reference instruments indicates that these units are not adversely affected by weather fluctuations (temperature or RH) or interfering pollutants.

### 3.2. Post-Deployment Co-location Period at Crestline

**Figure 5:**
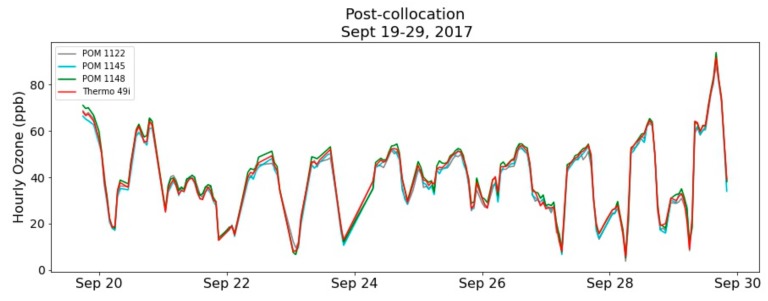
Post-deployment co-location time series for 1-h mean O_3_ concentrations.

**Figure 6:**
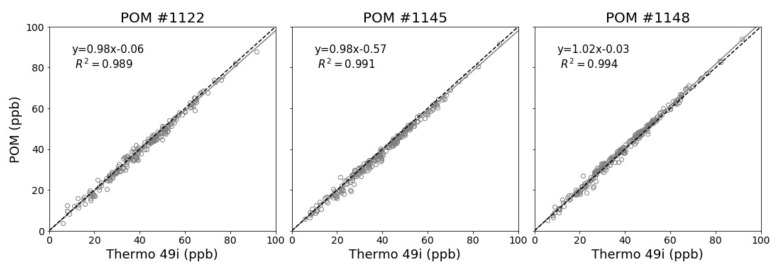
Post-deployment co-location correlation plots for 1-h O_3_ concentrations.

The post-deployment co-location at Crestline AMS took place for 10 days from 19–29 September 2017. The post-deployment co-location results provide a mechanism to verify that the POMs maintained their calibration and collected valid and accurate O_3_ measurements throughout the deployment period. Temperature conditions during the pre-deployment co-location ranged between 4 and 25 °C with a mean temperature of 13.3 ± 5.1 °C. The RH ranged between 13% and 99% with a mean RH of 54.3% ± 28.9%. The range for 1-h mean O_3_ concentrations experienced in the post-deployment co-location was 86 ppb with a maximum 1-h value of 91.6 ppb measured on September 29th by the Crestline AMS Thermo 49i. For hourly mean concentrations, R^2^ values were greater than 0.98 with slopes ranging from 0.98 to 1.02 and intercepts ranging from −0.03 to −0.57. The calculated MAE was less than 2 ppb with MBE calculated at −0.83, −1.24, and 1.30 ppb for POM 1122, 1145, and 1148, respectively. Figure 5 shows the time series for the post-deployment co-location and Figure 6 shows the scatter plots for the POMs vs. the Thermo 49i. These post-deployment co-location results indicate that the individual POMs maintained their calibration throughout the deployment period and collected accurate measurements with MAEs less than 2 ppb. Additionally, the performance was not adversely affected by changing weather conditions, interfering pollutants, or length of deployment.

### 3.3. Results from Deployment

**Figure 7:**
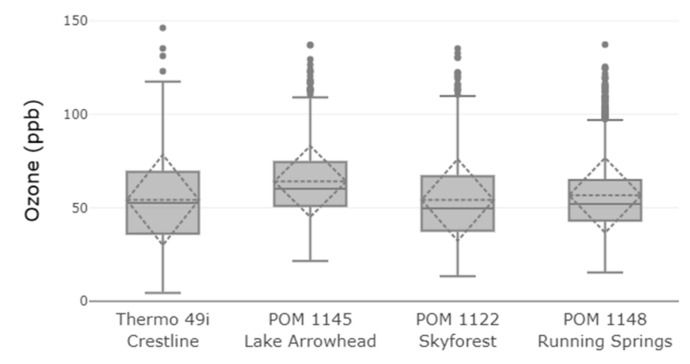
Box plots for the 1-h mean O_3_ concentrations for the four deployment locations.

The deployment of the sensors across the San Bernardino Mountains took place during the high O_3_ season for two months (17 July to 18 September 2017). Performing parallel monitoring during the high O_3_ season is critical for obtaining enough high concentration values to examine the relocation sites with the current monitoring site. Data recovery at the 1-h time average was found to be 99.9%, 96.5%, 73.4%, and 100% for the Thermo 49i, POM 1122, POM 1145, and POM 1148, respectively. POM 1145 in Lake Arrowhead, CA experienced a power outage due to an unforeseen water leak requiring the power outlet supplying the node to be turned off. As a result, data was not collected from 8:00 a.m. on 2 August 2017, to 4:00 p.m. on 18 August 2017, when the unit was outfitted with a solar panel, charge controller, and a 12-volt battery to provide power. Data rows with a missing value for any location were filtered out so a comparison between sites would have the same number of data points. After all rows with a missing value were dropped from further analysis, data recovery for the 1-h matched data was 70.4% (1032 rows) by which the four sites are characterized and compared. Temperature conditions for Crestline AMS during the deployment ranged from 8 to 34 °C with a mean temperature of 21.6 ± 4.8 °C. The RH ranged from 9% to 99% with a mean RH at 49.0% ± 18.5%. Table 3 provides the summary statistics, OLS regression statistics, and the mean measurement deviations calculated for the monitoring locations during the deployment period. The difference in mean O_3_ concentration between Crestline (54.2 ppb) and the three locations varied with Skyforest, CA (54.2 ppb) being identical, Running Springs, CA (56.7 ppb) being slightly higher on average, and Lake Arrowhead, CA (64.0 ppb) being about 10 ppb higher on average than the Crestline location. These spatial variations in O_3_ concentrations between locations could likely not have been predicted a priori without monitoring, highlighting the importance of developing less-expensive monitoring solutions to supplement the spatial resolution of current monitoring networks. The largest range of O_3_ concentrations was seen at the Crestline AMS which had the highest maximum and lowest minimum hourly concentration values. The summary statistics between the four locations are shown in Figure 7 by box plots for each of the sampling locations. The horizontal dotted line and dotted diamond indicate the mean and standard deviation of the sample. Note that the following figures and tables are ordered from left to right by distance from the Crestline AMS.

The OLS regression statistics for the 1-h matched data sets compare each of the potential relocation sites (*y*-axis) to the current Crestline AMS (*x*-axis) and provides insights into the similarity between the four locations. Immediately, the large intercept bias between the three locations stands out with intercepts at 28.8, 10.5, and 22.8 for Lake Arrowhead, Skyforest, and Runnings Springs, respectively. This intercept offset is primarily due to the nighttime differences between Crestline and the relocation sites likely caused by varying degrees of available local NO emission to scavenge O_3_. Regarding correlation, the three relocation sites correlate with Crestline with R^2^ values at 0.69, 0.80, and 0.57 for Lake Arrowhead, Skyforest, and Runnings Springs, respectively. Slope offsets between Crestline and the three relocation sites were found to be 0.65, 0.81, and 0.62 for Lake Arrowhead, Skyforest, and Runnings Springs, respectively. Of the three relocation sites, the Skyforest location most closely matches the Crestline location with the highest correlation and the smallest slope/intercept offset. If finding the location that most closely matches the diurnal trends of the Crestline AMS is required for relocation, then the Skyforest location would be the chosen relocation site as this site was found to have an identical mean O_3_ concentration throughout the deployment and regression statistics indicating the strongest commonality between Skyforest and the current monitoring site.

The MBD and MAD of the three locations with respect to Crestline provided insights on the spatial variability in O_3_ between locations. The MBD between Crestline and the three alternative locations was found to be 9.8, 0.0, and 2.5 ppb bias for Lake Arrowhead, Skyforest, and Running Springs, respectively. The MAD from Crestline was found to be 11.7, 8.3, and 12.1 ppb for Lake Arrowhead, Skyforest, and Running Springs, respectively. Looking at the MBD, the Skyforest location appears to be the most suitable location for relocation as this location matched the mean of the Crestline location. However, when examining the MAD, all three sites deviate from Crestline AMS with MAD > 8.0 ppb; indicating spatial variability between Crestline and these relocation sites. When separating the MAD between day and night hours, the predominant deviation between Crestline and the three locations takes place during nighttime hours. The MAD values for nighttime hours are 135%, 50%, and 83% higher than daytime MAD for Lake Arrowhead, Skyforest, and Running Springs, respectively. The cause for the increased nighttime deviation from the Crestline AMS is likely due to local factors affecting the titration of ozone between locations during nighttime conditions. Local factors including topography, populations, and traffic counts are discussed below to better understand these local factors and their impact on the spatial variation of O_3_ between Crestline and the relocation sites.

Since the Crestline AMS experiences some of the highest O_3_ concentrations in the SCAB, comparing the daily maximum O_3_ concentrations between the current monitoring site and the potential relocation sites is important to understand the difference between the locations with regard to daily 1-h maximum concentrations that could lead to exceedances of the 1-h standard. When considering relocating a site that experiences high O_3_ concentrations, care needs to be taken to ensure that the relocation site experiences O_3_ concentrations as high as or higher than the existing monitoring site. A data set of high values of the daily maximum 1-h O_3_ concentration was created and after filtering for the threshold value and missing data, 30 daily maximum values remained with data summarized in Table 4. The mean of the high values in the data set for Crestline, Lake Arrowhead, Skyforest, and Running Springs was 100.8, 106.9, 101.9, and 104.6 ppb, respectively. While each of the relocation sites experienced higher O_3_ concentrations than Crestline on average, only the Lake Arrowhead location provides a relocation site with a 95% confidence that the MBD would be greater than Crestline with a positive lower limit of MBD at 2.1 ppb, indicating that this location would likely be at least 2.0% higher than the Crestline AMS. Both Skyforest and Running Springs have negative values for the lower limit of the 95% CI of MBD at −2.6 and −1.5 ppb, respectively. These negative values indicate that these relocation sites, Skyforest and Running Springs, could potentially yield O_3_ concentrations lower than the current monitoring site by 2.6% and 1.5% respectively. If the requirement were set that the relocation site must on average experience higher concentrations than the existing site with a 95% CI on the MBD, then the Lake Arrowhead location would be chosen as the relocation site to meet this criterion. The regression statistics of the high values data set (*n* = 30) are similar in nature with the 1-h regression statistics (*n* = 1032) shown in the preceding section. Between the three relocation sites, Skyforest has the highest R^2^ value (0.70), slope nearest to one (0.90), and lowest intercept (11.3) which indicates this location most closely matches the diurnal trends experienced at Crestline.

**Figure 8:**
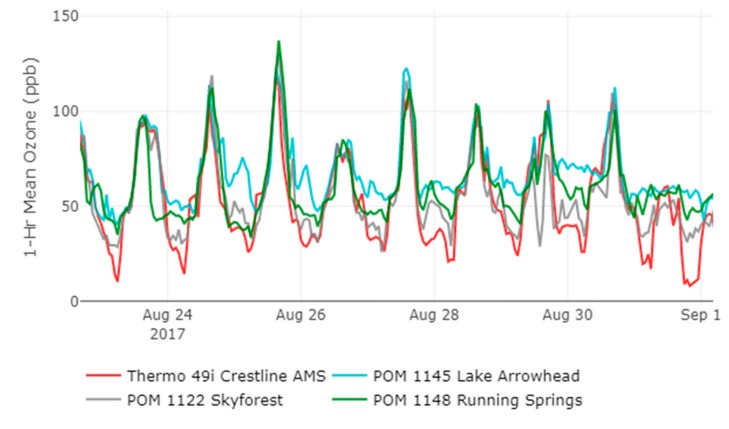
Timeseries for deployment, subset between 22 August and 1 September 2017.

**Figure 9:**
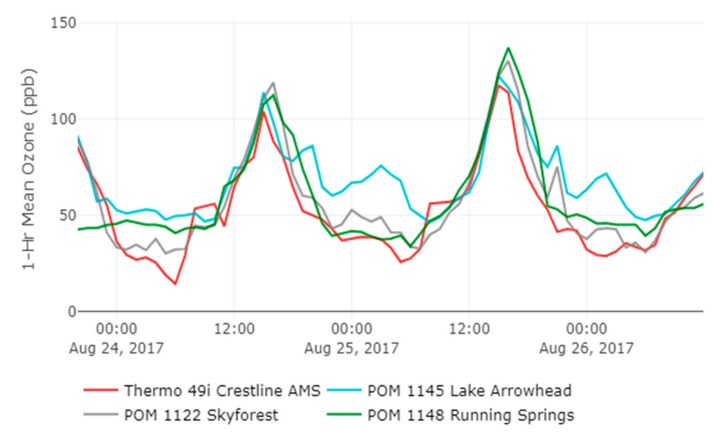
Time series for deployment subset between August 24 and 26, 2017.

The temporal differences between locations are shown in the time series plot shown in Figure 8 that is a subset from 21 August to 1 September 2017. The time series indicates that the three locations deviate from Crestline AMS predominantly during nighttime conditions when O_3_ concentrations are typically decreasing. The typical wind patterns of the region with daytime onshore winds blowing in from the west/southwest and daytime upslope flow for the mountains provide a steady source of O_3_ precursors for the elevated mountain communities. During the day, these upslope air masses are pushed up towards the boundary layer. In Figure 9, the timing of daily peak values between locations differs from the western sites (Crestline and Lake Arrowhead) peaking around 3 p.m. while the more eastern sites (Skyforest and Running Springs) peak an hour later around 4 p.m. During the evening time, wind patterns typically shift to an offshore direction with winds blowing from the northeast. These nighttime wind patterns lead to downslope air movement on the mountain which can lead to potential increases in O_3_ concentrations as polluted air masses near the boundary layer fall in elevation and pass through the mountain communities. An example of this can be seen in Figure 9 with O_3_ concentrations increasing in nighttime conditions on 25 August at 9 p.m. when hourly O_3_ concentrations increase by 10 and 15 ppb from the previous hour at Lake Arrowhead and Skyforest, respectively. A similar trend with regional-scale air flows and increasing nighttime O_3_ concentrations in mountain communities has been seen in the Front Range of the Colorado Mountains [32].

Comparing the four locations regarding the number of exceedances of the 2015 U.S. EPA 8-h O_3_ standard (70 ppb) provides another way for understanding the spatial variability of O_3_ between these locations. For the time-matched deployment data set, the Crestline location exceeded the 8-h O_3_ NAAQS standard 35 times while Lake Arrowhead, Skyforest, and Running Springs exceeded the standard 38, 27, and 28 times, respectively (Table 5). In comparison to Crestline, the Arrowhead location experienced six additional exceedance days, while Skyforest and Running Springs had five and four fewer days exceeding the standard, respectively. These differences indicate the spatial variability of O_3_ across the San Bernardino Mountains and provide an indication of how relocating the site may impact the number of 8-h exceedances recorded for the region. The O_3_ spatial variability with 10 additional days exceeding the standard at Lake Arrowhead in comparison with Skyforest is surprising as these two sites are located less than 5 km apart. This significant difference between locations in close proximity was surprising, not expected prior to monitoring, and could likely not have been predicted by simulation prior to monitoring. Many physical and chemical processes influence ambient O_3_ concentrations. Models that predict O_3_ concentrations simulate these physical and chemical processes. The simulation of atmospheric processes is challenging with the introduction of errors due to a lack of understanding of the physical and chemical processes, model assumptions, and data limitations [33]. Chemical process simulations include but are not limited to photolytic reactions and radical chemistry, while physical process simulations include but are not limited to emission sources and sinks, dispersion and diffusion, and meteorological conditions. An important physical process for air quality forecasting is the planetary boundary layer (PBL) which is the lowest layer of the atmosphere starting at the earth’s surface and capped by a stable layer [34]. The PBL layer height is difficult to predict when frontal boundaries (i.e., mountains) are present or multiple level thermal inversions are formed [35]. When the sun is setting, a second thermal inversion can form with the rapid loss of solar flux at the surface of the earth. This second layer forms the stable nocturnal boundary layer leaving a residual layer above that can potentially trap pollutants aloft. Simulations can be performed at varied spatial (regional to neighborhood) and temporal resolutions (yearly to hourly). The National Weather Service provides a national air quality forecast for the United States hour by hour at a spatial resolution of 12 km for O_3_ to provide advance notice of air pollution events [36]. In an active open-source development project, the U.S. EPA has developed the Community Multiscale Air Quality Modeling System (CMAQ) that consists of a suite of programs for creating air quality simulations [37]. The CMAQ model has been used to simulate air quality at finer spatial scales. With the addition of high-resolution input data, ozone concentrations were simulated for the Baltimore/Washington region at a 1 km spatial resolution. The bias between the simulation and surface ozone monitoring sites was found to follow a similar diurnal pattern with a positive mean bias in the early morning hours that decreases throughout the day until sunset when the bias starts increasing [38]. CMAQ was used in more complex topography in the San Joaquin Valley in California at 2–12 km and in the Colorado Front range at 4 km spatial resolution [39]. During O_3_ exceedances in Colorado, the simulation was found to capture the timing and rate of the initial rapid O_3_ production well, but largely underestimated the persistence of elevated concentrations when compared to surface O_3_ measurements. While the model correctly simulated regional O_3_ concentrations, verification with the local air monitoring stations revealed under- and over-estimation errors [32]. The spatial variability in O_3_ concentrations found between the locations in this study and the potential bias of O_3_ simulations indicates the importance of developing accurate sensing nodes and monitoring air pollutants in spatially dense networks to investigate the spatial variability of air pollutants and identify such spatial phenomena. This is especially true for regions with complex topography, meteorology, and atmospheric chemistry.

The topography and population of the distinct locations may have a factor in the differences in O_3_ concentrations between the four monitoring locations. The Crestline community has a population of roughly 10,000 residents with a valley topography with homes distributed around Lake Gregory and the Crestline AMS. The Lake Arrowhead and the Skyforest monitoring locations are at the outer southern edge of the populated Lake Arrowhead region (12,400 population) and are located along the SR-18 highway. Both Lake Arrowhead and the Skyforest location are higher in elevation by an estimated 350 m when compared to the Crestline location and have views looking into the lower San Bernardino County valley communities. This topography with an overlooking view is quite different than the valley topography of the Crestline location. Running Springs has a population of roughly 4800 residents with the monitor located at the southeastern edge of the community near an elementary school. The location of the Crestline AMS in the middle of a mountain community in contrast to the other three monitoring locations may potentially explain the differences in evening O_3_ concentrations. As commuters return home in the late afternoon/early evening, vehicle tailpipe emissions of NO titrate O_3_ from the Crestline community. Other stationary sources located within a mile of the Crestline AMS that may potentially play a role in O_3_ production and/or titration include two gasoline fuel stations, a wastewater treatment plant, and other establishments that may increase local traffic. The Crestline valley topography may also contribute to the stagnation of air which in turn leads to higher maxima during the day and lower minima concentrations at night. Prior research between urban and rural sites show similar trends seen in this work with a nighttime minimum for O_3_ more pronounced in urban locations [13]. These differences in topology, population, and siting location provide an explanation for why nighttime MAD is larger than daytime MAD between Crestline and the potential relocation sites.

**Figure 10:**
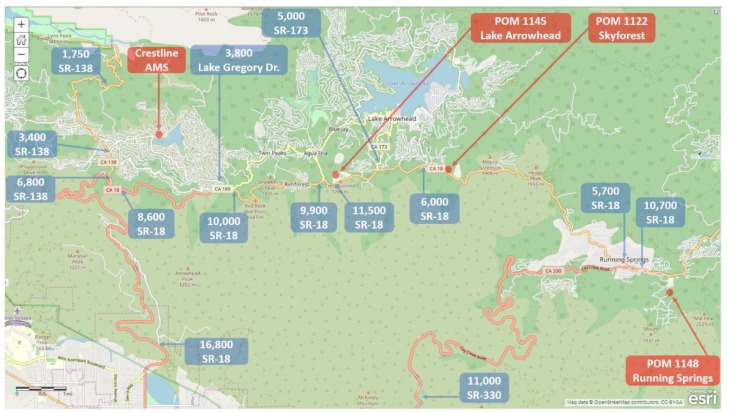
Annual Average Daily Traffic estimates by location for the monitoring region of interest. * SR: State Route.

The local traffic patterns in the region may also have an impact on O_3_ patterns and evening titration of O_3_ from the atmosphere. The California Department of Transportation (Caltrans) provides Annual Average Daily Traffic (AADT) estimates for state highways. AADT data for 2017 was retrieved from www.data.ca.gov as a geodatabase (GDB) with shapefiles for AADT which were viewed using ESRI ArcGIS Pro mapping software. Figure 10 shows the AADT estimates for relevant locations within the monitoring region. From the base of the mountains, AADT for California State Route 18 (SR-18) near Waterman Canyon is estimated at 16,800 daily counts. SR-18 is a common route for daily commuters who live in the mountain communities and work in the valley communities below. Roughly 40% of the AADT heading into the mountain communities on SR-18 diverts into Crestline via SR-138 with traffic counts estimated at 6800 counts for SR-138. The remaining 60% of the daily traffic continues along SR-18 towards Arrowhead with AADT estimated at 10,000 counts along SR-18 past Crestline. While Crestline has two significant corridors into the city from SR-18 with SR-138 and Lake Gregory Dr., Lake Arrowhead has four significant corridors into the community with Daley Canyon Rd to SR-189, SR-173, Arrowhead Villa Rd., and Kuffel Canyon Rd. These additional entrance/exit routes serve to spatially spread out the daily commuter traffic and may reduce the impact of the evening commuter traffic emissions scavenging O_3_. The SR-330 travels from the valley in San Bernardino and merges with SR-18 in Running Springs. SR-330 is the primary route for commuters heading into Running Springs and one of several routes that lead to the Big Bear region. AADT measured at the base of the mountain at SR-330 and Highland Ave. indicates AADT at the base of the mountain is estimated at 11,500. After the merging of SR-18 and SR-330 in Running Springs, SR-18 has increased traffic counts with AADT estimated at 10,700. This increased traffic flow on SR-18 with the merging with SR-330 in the Running Springs community contrasts with the Crestline monitoring location that is embedded in the community and located away from the SR-18.

The spatial scale of the Crestline AMS is considered to be a neighborhood scale monitoring site [19]. A neighborhood scale monitoring station is one that is defined to extend throughout an area of a city with relatively uniform land use with a range of 0.5 to 4.0 km [40]. The spatial variability of O_3_ between the Crestline AMS and the three sites (5.8 to 17.1 km from Crestline) supports this scale with the changing topography, population, and local land use between the mountain communities. When relocating a site, the data uses of the current site need to be examined to ensure the relocation site meets the desired data uses. The primary data uses for the Crestline O_3_ data are the evaluation of ambient air quality, protection of public health, and scientific research. Since Crestline is a site were high O_3_ concentrations are recorded in the SCAB, the evaluation of ambient air quality and determination of the NAAQS and CAAQS stands in the forefront. Therefore, the Lake Arrowhead location, which was the only site with a 95% CI on the MBD to be on at least 2% higher than the current site, would be the best choice for a relocation site. The Lake Arrowhead relocation option with higher O_3_ concentrations also suits the monitoring purpose of protecting public health as this location would likely indicate higher calculated AQI values and provide the associated AQI health messaging to warn residents during high O_3_ pollution events. Relocating to the Lake Arrowhead site would likely yield O_3_ monitoring data between 2.0% and 9.7% higher on average (95% confidence on MBD) than the Crestline AMS. This lack of continuity between measurement locations with higher concentrations at the relocation site would not be beneficial for the tracking of long-term air quality trends. Since the Lake Arrowhead relocation site would likely experience more O_3_ exceedances than the Crestline location, assessing the progress in achieving the air quality standards, with regard to regulatory or incentive actions taken to meet the standard, would not be measurable until several years of monitoring data is collected for trend tracking. Data uses involving long-term trend analysis and tracking of impacts on the environment and public health effects are benefited from long-term continuous measurements in one location. With that in mind, other than maintaining the site in Crestline, relocating to the Skyforest location would be the most likely relocation option since this site nearly matches the average concentrations at Crestline and most closely tracks the Crestline diurnal trends with the best regression statistics between the three relocation sites. With protecting public health and welfare as one of the ultimate goals of monitoring air quality, monitoring stations are strategically placed in locations with high population density. The current monitoring site in Crestline is the most strategically located as this location is situated near the center of a mountain community in contrast to the potential relocation sites located at the edges of their respective population centers.

In this study, the sensor nodes were developed to obtain O_3_ concentration across a region to determine potential alternative siting locations for an ambient air monitoring station with uncertainty around the renewal of the lease agreement for the current monitoring site. While these sensing nodes were purposely built for parallel monitoring of O_3_, the sensing platform could be used in other ambient applications due to the ease of installation, versatility with power and connectivity options, and accuracy of the O_3_ monitors. Each of the four locations monitored in this work included areas where the physical activity took place and ranged from water sports activities, high school athletics, biking/mountain biking, ice-skating, and softball/baseball. Two of the locations are adjacent to schools where physical education classes and school sporting events are conducted. Deployment of real-time O_3_ sensors in O_3_ pollution impacted communities at schools could provide data to school administrators and coaches on their current hyper-local O_3_ concentrations that could be used to make determinations on the appropriateness of conducting physical activities. Threshold values based on health and exposure studies could be established to set up alert notifications to inform decision-makers when O_3_ concentrations reach unhealthy or unsafe conditions.

This evaluation of the spatial and temporal ozone trends is limited due to the specific application of this project to determine if a nearby location to the current Crestline AMS could serve a potential relocation site. This study was performed in a relatively tight geographical area and over a 2-month period during the high ozone season. A more comprehensive study to investigate spatial and temporal trends in the region could be designed and would include additional sites (urban/rural) and be performed for a longer duration to include multiple seasons.

## 4. Conclusions

This paper presents the development and deployment of a small network of a highly accurate remote O_3_ sensor nodes for performing parallel monitoring to examine three potential relocation sites for a regulatory air monitoring site. The deployment methodology of the three O_3_ sensing nodes included a pre-deployment co-location calibration to a reference O_3_ analyzer with post-deployment co-location results indicating a MAE for 1-h O_3_ concentrations to be less than 2 ppb between the POMs and the O_3_ reference instrument at the monitoring site. The O_3_ sensing nodes provided accurate, precise, and real-time O_3_ measurements that were displayed on an online dashboard for real-time viewing and reporting. The high-level of confidence in the data generated by these sensing nodes allows for investigating the spatial and temporal trends across the distinct locations that could serve as a relocation site for the current regulatory monitoring station in the San Bernardino Mountains. The results indicate that spatial variability exists between these locations with differences more pronounced in the evening hours. When examining exceedances of the 2015 8-h standard at 70 ppb, locations within 5 km from each other differed by more than 10 exceedance days over the deployment period. The parallel monitoring was successful in providing the data to adequately defend a relocation strategy for the current O_3_ monitoring site with only one site providing a 95% confidence that concentrations would be higher than the current monitoring location.

## Figures and Tables

**Table 1:** Federal and State standards for ozone (obtained September 2019).

Standard for Ozone	1-h Average	8-h Average (Year Established)
National Ambient Air Quality Standard	120 ppb (1979)	70 ppb (2015) 75 ppb (2008) 80 ppb (1997)
California Ambient Air Quality Standard	90 ppb	-

**Table 2:** Deployment dates and number of days per deployment period.

Period	Dates	# of Days
Pre-deployment co-location	7/11/17 to 7/19/17	8
Deployment	7/19/17 to 9/19/17	62
Post-deployment co-location	9/19/17 to 9/29/17	10

**Table 3:** Summary information and 1-h statistics for the four monitoring locations.

Location	Crestline	Lake Arrowhead	Skyforest	Running Springs	
Instrument	Thermo 49i	POM 1145	POM 1122	POM 1148	Units
Population	10,700	12,400	300	4800	no. residents
Elevation	1390	1753	1733	1858	m
Distance from Crestline AMS	0	5.8	9.5	17.1	km
**Statistics (1-h average)**					
Mean Ozone Conc.	54.2	64.0	54.2	56.7	ppb
Standard Deviation	24.2	18.9	21.8	20.0	ppb
Minimum Conc.	4.5	21.5	13.4	15.4	ppb
Maximum Conc.	146.2	137.1	135.2	137.3	ppb
Hourly data points	1032	1032	1032	1032	count
Slope	-	0.65	0.81	0.62	-
Intercept	-	28.8	10.5	22.8	-
R^2^	-	0.69	0.80	0.57	-
Mean Bias Deviation (MBD)	-	9.8	0.0	2.5	ppb
Mean Absolute Deviation (MAD)	-	11.7	8.3	12.1	ppb
MAD—Daytime		7.1	6.7	8.7	ppb
MAD—Nighttime		16.7	10.0	15.9	ppb

**Table 4:** Summary Statistics and 95% confidence interval for the daily 1-h maximum O_3_ concentration.

Location	Crestline	Lake Arrowhead	Skyforest	Running Springs	
Instrument	Thermo 49i	POM 1145	POM 1122	POM 1148	Units
Mean Conc.	100.8	106.9	101.9	104.6	ppb
MBD	-	6.08	1.08	3.81	ppb
SD MBD	-	8.89	8.29	11.93	ppb
Lower limit of MBD (95% CI)	-	2.10	−2.63	−1.53	ppb
Upper Limit of MBD (95% CI)	-	10.05	4.79	9.15	ppb
Lower Limit %	-	2.0	−2.6	−1.5	%
Upper Limit %	-	9.7	4.7	8.9	%
Slope	-	0.61	0.90	0.63	-
Intercept	-	45.7	11.3	41.5	-
R^2^	-	0.59	0.70	0.40	-

**Table 5:** Exceedances of the U.S. EPA 2015 8-h ozone standard of 70 ppb.

Location—Unit	No. of Exceedances (Days)
Crestline—Thermo 49i	32
Skyforest—POM 1122	27
Lake Arrowhead—POM 1145	38
Running Springs—POM 1148	28

## Supplementary Materials

The following are available online at https://www.mdpi.com/1424-8220/20/1/16/s1, Figure S1: 2B Tech Personal Ozone (O_3_) Monitor (POM), Figure S2: Netronix Thiamis 1000, Table S1: Bill of Materials for O_3_ Sensing Node, Figure S3: Calibration Verification of 2B POMs, Figure (S4): Map of Southern California with deployment locations, Data Analysis/Calculations section with Equation (S1): Mean Bias Error (MBE), Equation (S2): Mean Absolute Error (MBE), Equation (S3): Mean Bias Deviation (MBD), and Equation (S4): Mean Absolute Deviation (MBD), a section with Hampel Filter information, High Values Data Set Equations with Equation (S5): Lower Limit of 95% CI (L), Equation (S6): Upper Limit of 95% CI (U), Equation (S7): Lower Limit %, and Equation (S8): Upper Limit %, and Table S2: In-situ collocation calibration offsets based on ordinary least squares regression.
