# Supplementary material for: Development of a Network of Accurate Ozone Sensing Nodes for Parallel Monitoring in a Site Relocation Study
Source: Sensors (Basel). 2019 Dec 18;20(1):16. doi: 10.3390/s20010016 (PMC6982912; doi:10.3390/s20010016)
Supplement: Supplementary file 1 [file sensors-20-00016-s001.pdf]

Supplemental Information

Development of a Network of Accurate Ozone Sensing Nodes for Parallel Monitoring in a Site Relocation Study

Brandon Feenstra[1, 2, 3, *], Vasileios Papapostolou[1], Berj Der Boghossian[1], David Cocker[2, 3] and Andrea Polidori[1, *]

1 South Coast Air Quality Management District, Air Quality Sensor Performance Evaluation Center (AQ-SPEC), Diamond Bar, CA 91765, USA; vpapapostolou@aqmd.gov (V.P.); bderboghossian@aqmd.gov (B.D.)

2 Department of Chemical & Environmental Engineering, University of California-Riverside, Riverside, CA 92521, USA; dcocker@engr.ucr.edu (D.C.)

3 Bourns College of Engineering, Center for Environmental Research and Technology (CE-CERT), University of California-Riverside, Riverside, CA 92507, USA

* Correspondence: bfeenstra@aqmd.gov (B.F.); apolidori@aqmd.gov (A.P.); Tel.: +1-909-396-2193 (B.F.); Tel.: +1-909-396-3283 (A.P.)

Received: 1 November 2019; Accepted: 15 December 2019; Published: date

Supplemental Information (SI)

1. Figure S1. 2B Tech Personal Ozone (O_3) Monitor (POM).
2. Figure S2. Netronix Thiamis 1000
3. Table S1. Bill of Materials for O_3 Sensing Node
4. Figure S3. Calibration Verification of 2B POMs
5. Figure S4. Map of Southern California with deployment locations
6. Data Analysis / Calculations
   Equation S1. Mean Bias Error (MBE)
   Equation S2. Mean Absolute Error (MBE)
   Equation S3. Mean Bias Deviation (MBD)
   Equation S4. Mean Absolute Deviation (MBD)
7. Hampel Filter
8. High Values Data Set Equations
   Equation S5. Lower Limit of 95% CI (L)
   Equation S6. Upper Limit of 95% CI (U)
   Equation S7. Lower Limit %
   Equation S8. Upper Limit %
9. Table S2: In-situ collocation calibration offsets based on ordinary least squares regression

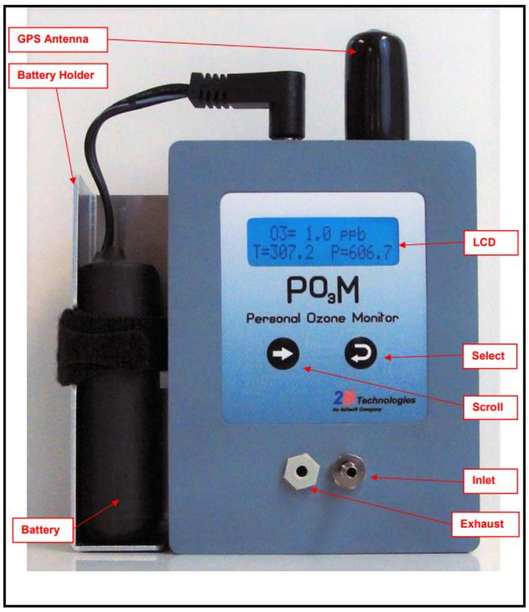

Figure S1. 2B Tech Personal Ozone Monitor (POM). **Source:** https://twobtech.com/docs/manuals/model_POM_revF-4.pdf.

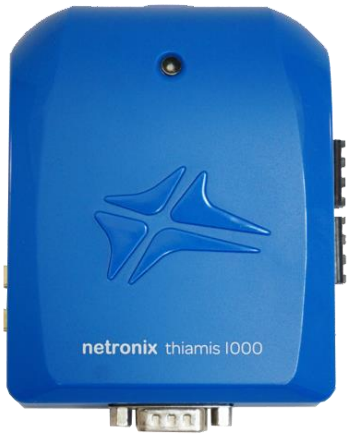

Figure S2. Netronix Thiamis 1000.

Table S1. Bill of Materials for O_3 Sensing Node.

Materials	Manufacturer	Part number	Est. Cost (USD)
POM	2B Technologies	MODELPOM	$5,000
Filter housing	2B Technologies	FILTERHS (25mm)	$150
Thiamis 1000	Netronix	T1K	$1,000
Enclosure + pole mount kit + back panel kit	Fibox	AR14127CHSSL	$100
Vent	Attabox	AH-V60	$10
120V to 12V converter	Aiposen	S-60-12	$15
50-watt solar panel	Renogy	RNG-50D	$75
Charge controller	Renogy	CTRL-PWM10	$20
12V 15 AHR Battery	Bioenno	BLF-1215AS	$130
**Total Est. Cost:**			**$6,500**

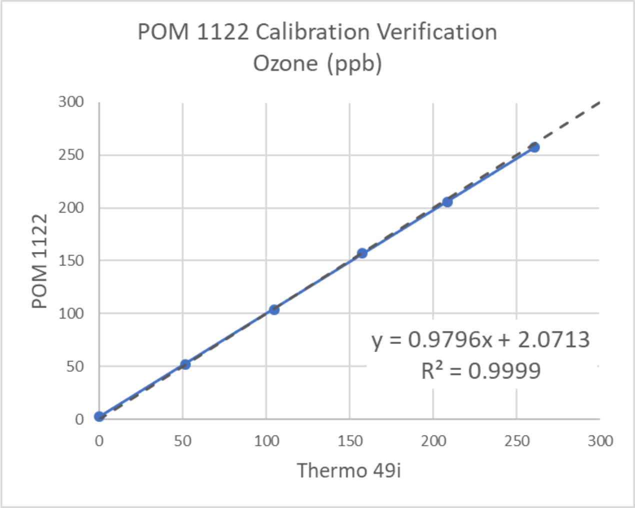

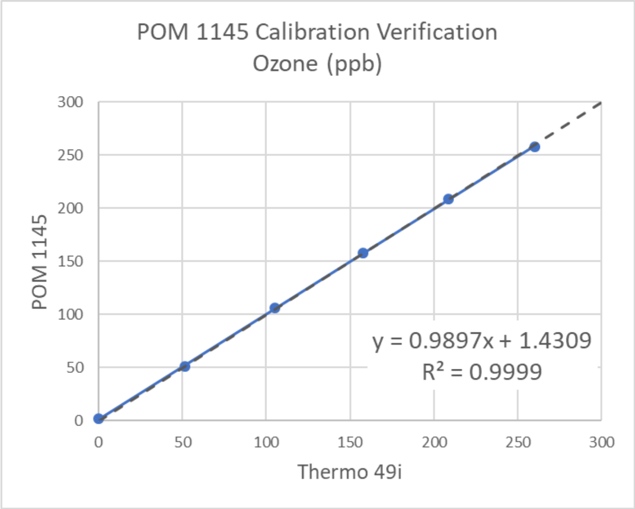

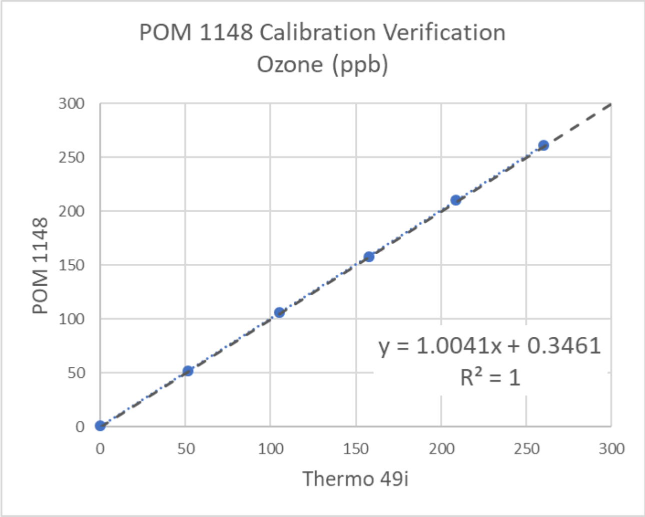

Figure S3. Calibration Verification of 2B POMs.

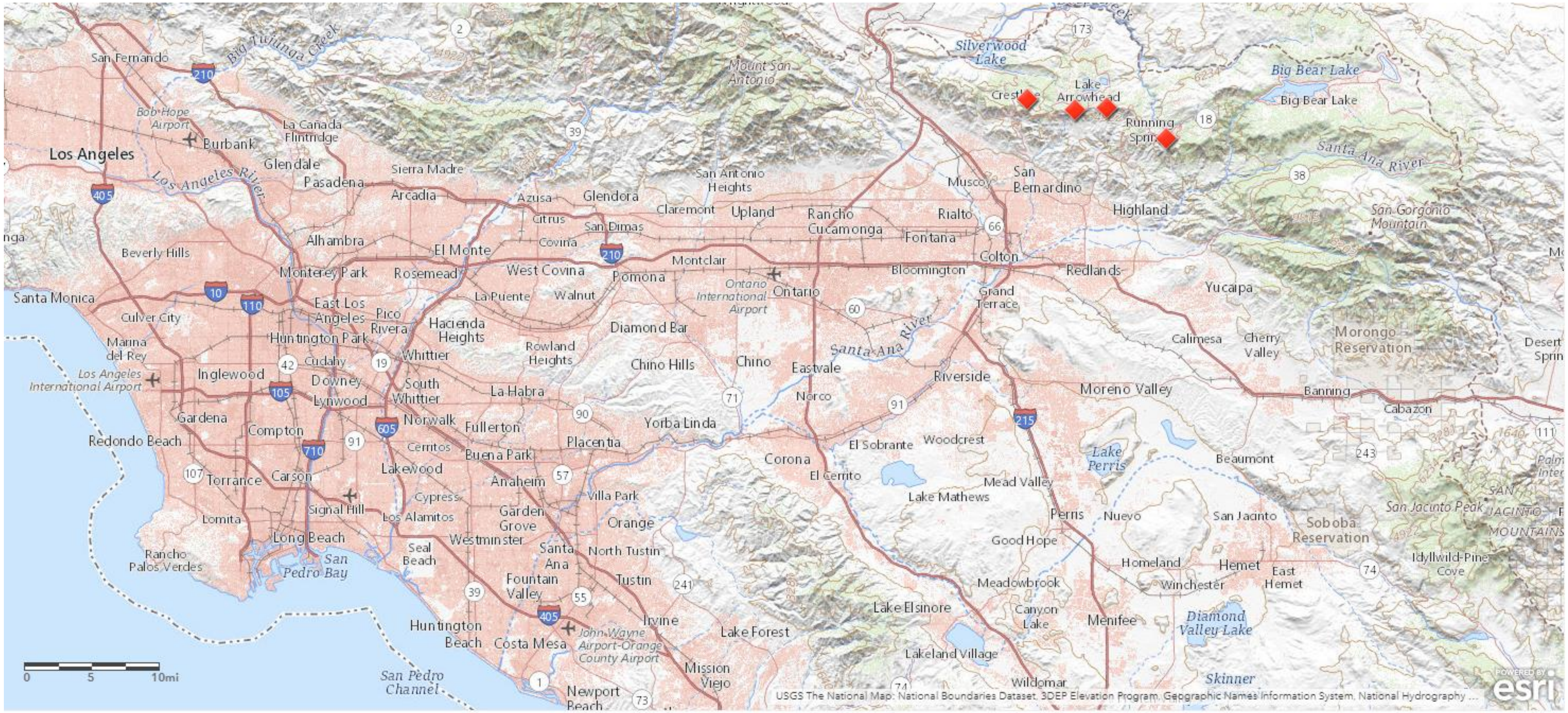

Figure S4. Extended Map of Southern California with Deployment Locations.

Calculations

Mean Bias Error (MBE) and Mean Absolute Error (MAE) calculate the measurement error by examining the hourly differences between the 2B POM and the Crestline AMS Thermo 49i O_3measurement. The MBE between the sensor and the reference O_3 instrument provides a metric that indicates the tendency of the sensor to either under- or over-estimate the reference O_3 concentrations during the pre- and post-deployment collocation periods. The units of both MBE and MAE are calculated in ppb, which is identical to the units of measurement. Care must be taken with the MBE statistic, as over-estimated errors will cancel out under-estimated errors in the calculation of the bias error. The MAE provides a better metric for actual measurement error between sensor and reference. The equations for MBE and MAE are found in equation S1 and S2, respectively.

$$\text{Equation S1} \qquad \text{Mean Bias Error (MBE)} = \frac{1}{n}\sum_{i=1}^{n} (X_i - X_t) \qquad (1)$$

$$\text{Equation S2} \qquad \text{Mean Absolute Error (MAE)} = \frac{1}{n}\sum_{i=1}^{n} |(X_i - X_t)|, \qquad (2)$$

where,

X_i is the 1-hr average measurement provided by the low-cost sensor
X_t is the 1-hr average measurement provided by the Crestline Thermo 49i
n is the number of 1-hr time-matched data pairs

Mean Bias Deviation (MBD) and Mean Absolute Deviation (MAD) calculate the deviation in O_3 concentrations between deployment locations by examining the hourly differences between the POM unit and the Crestline AMS Thermo 49i O_3measurements. The MBD between the POM and Crestline provides a metric that indicates the tendency of a relocation site to either under- or over-estimate O_3concentrations when compared to the Crestline AMS. The equations for MBD and MAD are found in equation S3 and S4, respectively.

$$\text{Equation S3} \qquad \text{Mean Bias Deviation (MBD)} = \frac{1}{n}\sum_{i=1}^{n} (X_i - X_t) \qquad (3)$$

$$\text{Equation S4} \qquad \text{Mean Absolute Deviation (MAD)} = \frac{1}{n}\sum_{i=1}^{n} |(X_i - X_t)|, \qquad (4)$$

where,

X_i is the 1-hr average measurement provided by the POM
X_t is the 1-hr average measurement provided by the Crestline Thermo 49i
n is the number of 1-hr time-matched data pairs

Hampel Filter

The Hampel Filter function applies a filter along a rolling sample of an input vector. The median and standard deviation of the median are computed for the rolling sample window. Points that exceed a set threshold for the standard deviation of the median of the rolling sample window are characterized as outliers and replaced with the rolling median value. The sample window consists of ten data points which would constitute 10 min of data. If a sample value differed from the rolling median by more than six standard deviations, the sample value was replaced with the median value for the rolling window.

95. % Confidence Interval Calculations

These calculations are adapted from the California Air Resources Board's (CARB's) Air Monitoring Technical Advisory Committee (AMTAC) document that provides guidelines for site relocation and Parallel Monitoring.

$$\text{Equation S5} \qquad \text{Lower Limit of 95\% CI (L)} = \bar{d} - \left(t * \frac{s}{\sqrt{n}}\right) \qquad (5)$$

$$\text{Equation S6} \qquad \text{Upper Limit of 95\% CI (U)} = \bar{d} + \left(t * \frac{s}{\sqrt{n}}\right), \qquad (6)$$

where,

$\bar{d}$ = Mean Bias Deviation (Eq. S3)
s = standard deviation of the MBD
n = number of matching pairs
df = degree of freedom = n-1
t = T score found in from at T-score distribution table, specific to degree of freedom and confidence interval level of certainty

$$\text{Equation S7} \qquad \text{Lower Limit } \% = \frac{L}{(\bar{X} + \bar{Y})/2} * 100 \qquad (7)$$

$$\text{Equation S8} \qquad \text{Upper Limit } \% = \frac{U}{(\bar{X} + \bar{Y})/2} * 100, \qquad (8)$$

where,

L = Lower Limit of Confidence Interval (Eq. S5)
U = Upper Limit of Confidence Interval (Eq. S6)
$\bar{X}$ = Mean of Existing Monitoring Site
$\bar{Y}$ = Mean of Relocation Site

Ordinary Least Squares Calibration Equations

POM #	Slope offset	Intercept offset
1122	1.066	-1.566
1145	1.002	0.679
1148	1.039	1.207

Table S2. In-situ collocation calibration offsets based on ordinary least squares regression.
